# Supplementary material for: Functionalization of PET with Phosphazene Grafted Graphene Oxide for Synthesis, Flammability, and Mechanism
Source: Materials (Basel). 2021 Mar 17;14(6):1470. doi: 10.3390/ma14061470 (PMC8002576; doi:10.3390/ma14061470)
Supplement: Supplementary file 1 [file materials-14-01470-s001.pdf]

materials

MDPI

Supplementary Material

Functionalization of PET with Phosphazene Grafted Graphene Oxide for Synthesis, Flammability, and Mechanism

Lifei Wei [1,2], Rui Wang [2,*], Zhiguo Zhu [2], Wenqing Wang [2] and Hanguang Wu [2]

[1] Polymer Research Institute, Sichuan University, No.24 South Section 1, Yihuan Road, Chengdu 610065, China; wlf13581518318@163.com

[2] School of Material Science and Engineering, Beijing Institute of Fashion Technology, No. A2, East Yinghua Street, Chaoyang District, Beijing 100029, China; clyzzg@bift.edu.cn (Z.Z.); 20180021@bift.edu.cn (W.W.); 20180064@bift.edu.cn (H.W.)

* Correspondence: clywangrui@bift.edu.cn; Tel.: +86-010-64288279

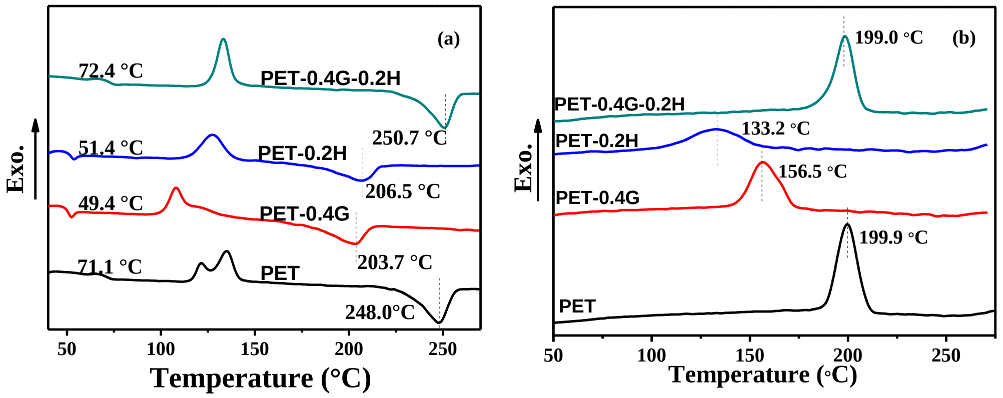

Figure S1. Differential scanning calorimetry (DSC) curve of neat PET and its composites: (**a**) heat curve; (**b**) cool curve.

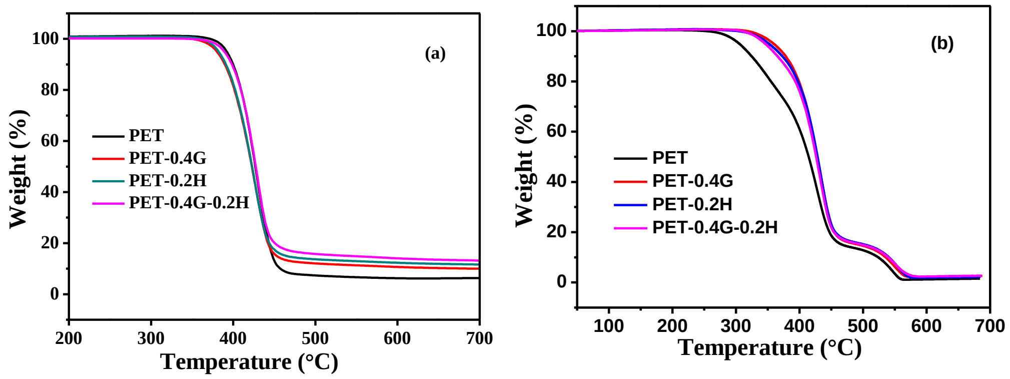

Figure S2. Thermogravimetric analysis (TGA) curve of neat polyethylene terephthalate (PET) and its composites: (**a**) N_2; (**b**) Air.

Publisher's Note: MDPI stays neutral with regard to jurisdictional claims in published maps and institutional affiliations.

Copyright: © 2021 by the authors. Licensee MDPI, Basel, Switzerland. This article is an open access article distributed under the terms and conditions of the Creative Commons Attribution (CC BY) license (http://creativecommons.org/licenses/by/4.0/).

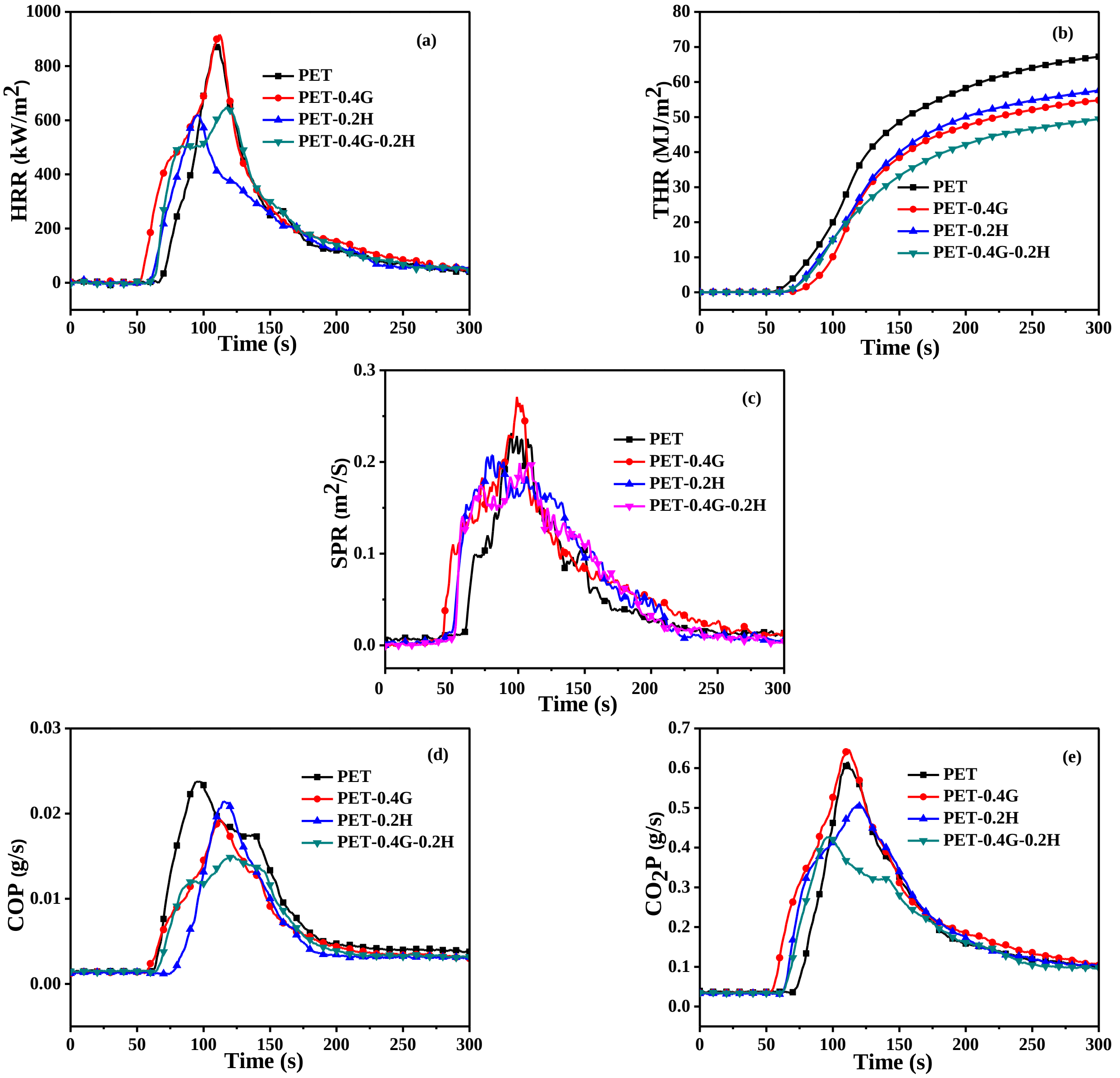

Figure S3. (**a**) Heat release rate, (**b**) total heat release, (**c**) smoke production rate, (**d**) CO production and (**e**) CO_2 production versus time curves of neat PET and its composites.
